# Modeling post‐translational modifications and cancer‐associated mutations that impact the heterochromatin protein 1α‐importin α heterodimers

**DOI:** 10.1002/prot.25752

**Published:** 2019-06-14

**Authors:** Michael T. Zimmermann, Monique M. Williams, Eric W. Klee, Gwen A. Lomberk, Raul Urrutia

**Affiliations:** ^1^ Bioinformatics Research and Development Laboratory, and Precision Medicine Simulation Unit, Genomic Science and Precision Medicine Center (GSPMC) Medical College of Wisconsin Milwaukee Wisconsin; ^2^ Clinical and Translational Sciences Institute Medical College of Wisconsin Milwaukee Wisconsin; ^3^ Department of Biochemistry Mayo Clinic Rochester Minnesota; ^4^ Division of Biomedical Statistics and Informatics Mayo Clinic Rochester Minnesota; ^5^ Division of Research, Department of Surgery Medical College of Wisconsin Milwaukee Wisconsin; ^6^ Department of Pharmacology and Toxicology Medical College of Wisconsin Milwaukee Wisconsin; ^7^ Genomic Science and Precision Medicine Center (GSPMC) Medical College of Wisconsin Milwaukee Wisconsin; ^8^ Department of Biochemistry Medical College of Wisconsin Milwaukee Wisconsin

**Keywords:** cancer, CBX5, functional genomics, HP1α, molecular modeling

## Abstract

Heterochromatin protein 1α (HP1α) is a protein that mediates cancer‐associated processes in the cell nucleus. Proteomic experiments, reported here, demonstrate that HP1α complexes with importin α (IMPα), a protein necessary for its nuclear transport. This data is congruent with Simple Linear Motif (SLiM) analyses that identify an IMPα‐binding motif within the linker that joins the two globular domains of this protein. Using molecular modeling and dynamics simulations, we develop a model of the IMPα‐HP1α complex and investigate the impact of phosphorylation and genomic variants on their interaction. We demonstrate that phosphorylation of the HP1α linker likely regulates its association with IMPα, which has implications for HP1α access to the nucleus, where it functions. Cancer‐associated genomic variants do not abolish the interaction of HP1α but instead lead to rearrangements where the variant proteins maintain interaction with IMPα, but with less specificity. Combined, this new mechanistic insight bears biochemical, cell biological, and biomedical relevance.

## 1 INTRODUCTION

Three known HP1 isoforms, HP1α (CBX5), HP1β (CBX1), and HP1γ (CBX3), are critical for regulating gene expression networks that are crucial for normal embryonic development^1^ and cancer associated processes, including differentiation,^2^, ^3^ cell proliferation,^4^, ^5^ cell cycle control,^4^, ^6^, ^7^, ^8^, ^9^ apoptosis,^8^, ^10^ and DNA damage response.^11^, ^12^, ^13^, ^14^, ^15^, ^16^, ^17^, ^18^ In fact, a conservation in sequence, structure, and function among these proteins in organisms ranging from *Drosophila melanogaster* to human reflects the biological importance and biomedical implications of their alterations. Alterations in HP1 proteins range from overexpression, somatic mutations, CNVs, and abnormalities in the function of their upstream regulators. Early studies performed by our group and others have demonstrated that signaling‐mediated mechanisms, yet to be fully understood, lead to the extensive post‐translational modifications of HP1 proteins. Some of these modifications fall in the globular domains of these proteins; the globular domains read histone marks and are responsible for homodimerization and heterodimerization. These domains are joined by an instrinsically disordered region known as the linker, which serves as an integrator of phosphorylation‐mediated signaling events. However, some of the detailed structural‐functional relationships of the linker domain are by far less understood than those of the globular domains. Thus, the current study uses the HP1α linker domain, as a model for extending our understanding of how these proteins can be regulated via the linker domain through heterodimerization events that ultimately lead to the translocation of this protein to the nucleus, the cellular region where it is functionally needed. Moreover, we model how post‐translational modifications, deposited in the linker region in response to upstream regulators such as cancer‐associated mutations, alter linker structure and thereby bonding patterns during interphase, likely impacting import and the downstream pathway.

Our findings indicate that post‐translational modifications of the HP1α linker are a critical factor in altering interaction with IMPα, and that genomic variants alter the pattern of interactions making them less specific. We used computational molecular modeling and dynamics simulation, and structural bioinformatics tools to significantly expand our knowledge of the intramolecular and intermolecular behavior of HP1α in direct relation to biological processes that are crucial for the maintenance of genomic integrity. This work significantly extends our understanding of the dynamic behavior of HP1α and how it achieves intermolecular interactions outside of the chromoshadow domain. Additionally, our approach to use multiple computational measures that help to inform one another emphasizes that each metric should not be interpreted in isolation; they should be jointly considered to better understand what they indicate about the molecule. Most importantly, we have gained insight for how HP1α alterations may disrupt signals relayed through mitogenic signaling pathways and heterochromatin regulation during each cell cycle. Thus, these results must be taken into consideration as mechanisms that are likely to influence the function of HP1 proteins during development, homeostasis control, and disease.

## 2 MATERIALS AND METHODS

### 2.1 Multiple sequence alignment

Protein sequences for the human and mouse HP1 family members, HP1α (CBX5), HP1γ (CBX3), and HP1β (CBX1) were downloaded from UniProt^19^ and aligned to each other using standard parameters of Clustal‐Omega^20^ at the European Bioinformatics Institute. Amino acid equivalences between isoforms used in molecular modeling were taken from the multiple sequence alignment (MSA). The sequence identity of the proteins was assessed by pairwise alignment via the EMBOSS‐Needle open‐source software platform.^21^ HP1α and HP1β demonstrated 76.9% residue similarity, while HP1α and HP1γ share 66.7% residue similarity. The MSA is available in Table S1 and was used in the construction of the protein models.

### 2.2 Linear motif analysis for nuclear localization signal and phosphorylation site prediction

The canonical binding mode of the nuclear importin receptor protein, IMPα, and nuclear localization signal (NLS) containing proteins was explored in the PDB and the literature.^22^, ^23^, ^24^, ^25^, ^26^, ^27^, ^28^, ^29^, ^30^ The sequence of HP1α was examined for NLS motifs using the prediction algorithms of PSORTII^31^ and cNLS Mapper.^32^, ^33^ Both algorithms identified a bipartite NLS in the HP1α linker, as previously predicted.^34^ Potential phosphorylation sites in the HP1α linker and the kinases that catalyze these modifications were identified using several in silico predictive servers including: NetPhos 3.1,^35^, ^36^ Kinase Phos 2.0,^37^ DISPHOS 1.3,^38^ GPS 3.0,^39^ and PhosphoSVM.^40^ The predictions were cross‐referenced, and experimentally validated sites were assessed via PhosphositePlus^41^ and PHOSIDA.^42^, ^43^ We also annotated the protein sequence using standard metrics and sequence‐based linear motifs from multiple tools: InterProScan,^44^ MESSA,^45^ VADAR,^46^ Molprobity,^47^, ^48^, ^49^ DisEMBL,^50^ IUPred2A,^51^ and OnD‐CRF.^52^

### 2.3 Modeling of the HP1α‐IMPα complex

The crystal structure of the linker peptide which connects the chromodomain and chromoshadow domain has not been solved, likely due to its flexibility. Therefore, we built the model of the peptide using the sequence of residues 87‐109 and the Builder Function in Discovery Studio^53^ linking the chromodomain and chromoshadow domain. The sequence ID and 3D structures utilized to generate our models can be found in Table S2. We used PyMol^54^ for molecular visualization.

The binding mode of a bipartite NLS to IMPα was previously demonstrated for the histone‐binding protein N1N2 in *Xenopus laevis* 55 and now serves as a standard prototype of this intermolecular association. The experimental structure for N1N2 NLS was downloaded (PDB: 1PJN^55^) and used in threading to produce a model of the HP1α linker bound to IMPα. We compared our threaded model to the results of full‐length HP1α docking to IMPα via ClusPro.^56^, ^57^, ^58^, ^59^, ^60^

### 2.4 Immunoprecipitation and mass spectrometry

HP1α immunoprecipitation was performed from HeLa cells. An antibody to HP1α (Abcam, ab77256) or control IgG was conjugated to Protein A/G Magnetic Beads (ThermoFisher Scientific) through disuccinimidyl suberate (DSS) crosslinking. Cells were lysed with immunoprecipitation Lysis/Wash Buffer (Thermo Scientific‐Pierce), and lysates were incubated with the conjugated antibodies overnight at 4°C. Immunoprecipitated complexes were washed, eluted and run on a 4%‐15% Criterion Tris‐HCl Protein Gel (Bio‐Rad), which was subsequently visualized with BioSafe Coomassie Stain (Bio‐Rad). Each gel lane was divided into eight sections, destained, dehydrated, dried, and subjected to trypsin digestion. Liquid chromatography (LC)‐ESI‐MS/MS analysis was then performed on a Thermo Scientific LTQ Orbitrap mass spectrometer at the Mayo Clinic Proteomics Core.

### 2.5 Modeled variants

We modeled experimentally relevant phosphorylated, nonphosphorylatable mutant, or phosphomimetic mutant forms of S92, S95, S97, and S103 within the HP1α linker separately or in combination. As controls for binding, HP1α NLS binding mutants were also generated by mutating each cluster of basic residues within the bipartite sequence to acidic residues. Additionally, rare single nucleotide variants in HP1α that are rare in the currently healthy adult population (GnomAD minor allele frequency, MAF, ≤1 × 10^−4^) were mined from The Cancer Genome Atlas (TCGA),^61^ the Catalogue Of Somatic Mutations In Cancer (COSMIC),^62^ and the Exome Aggregation Consortium (ExAC) or GnomAD^63^ (see Table S3). The cancer‐related variants were then assessed for degree of pathogenicity using multiple sequence‐based algorithms: Mutation Taster2,^64^ Polyphen‐2,^65^ MutPred2,^66^ and SIFT.^67^ These algorithms did not provide equivalent annotations for each variant, (Table S4 and without any previous functional validation, variants with conflicting annotations were considered variants of uncertain significance (VUS). Computational mutagenesis was performed in Discovery Studio.^53^ Variants are listed in Table S5.

### 2.6 Molecular dynamics simulations

Molecular dynamics (MD) simulations were executed as in our previous studies.^68^ Briefly, simulations for each condition were run in NAMD using the Charmm36 all‐atom force‐field^69^ and a 1 fs integration time‐step. The backbone C^α^ atoms of IMPα were constrained using harmonic restraints. Models were simulated in the Generalized Born Molecular Volume (GBMV) implicit solvent model in Discovery Studio^53^ at a dielectric constant of 80 and pH 7.4. Energy minimization commenced for 2000 steps with steepest descent. Hydrogen bonds were constrained using the SETTLE algorithm. Independent triplicate systems were initialized and heated to 300 K over 600 ps to mimic physiological conditions, equilibrated for 400 ps with subsequent production run for 10 ns (30 ns total for each condition).

### 2.7 Statistical analysis

Prior to calculations, all trajectories were superimposed using C^α^ atoms of IMPα. We calculated root‐mean‐square deviation (RMSD) and root‐mean‐square‐fluctuation (RMSF) values to infer the flexibility and mobility of the peptides throughout the simulation and using C^α^ atoms. The surface area of the IMPα receptor alone and the peptide alone were measured to uncover any differences in conformation or folding, and then buried solvent accessibility surface area (SASA) of the peptide in each condition was measured and plotted. Hydrogen bonds and salt bridges between the linker peptide and IMPα were calculated across the conditions and visualized as heat maps. We analyzed hydrogen bonds that had a frequency ≥ 0.5 in at least three different conditions. The energy of interaction, encompassing van der Waals and electrostatic forces, was calculated to survey the interaction strength of each complex. The C^α^ atoms of residue pairs involved in hydrogen bonding or salt bridge interactions between the peptide and IMPα were selected for distance metrics as simplified measures of interaction. Contact maps defining the intermolecular contacts between the HP1α peptide across the trajectory were generated. Atoms were considered contacting if they were within 3.9 Å and the fraction of simulation time spent in contact was recorded. Principle component analysis (PCA) of the C^α^ atoms was calculated in Cartesian space. All distributions were compared using permutation and *t* tests, as previously described.^68^

### 2.8 Software

Initial protein models were constructed in Discovery Studio v2017^53^ and simulations completed directly through NAMD^70^ version 2.12. The bio3d^71^ R package version 2.2.4 was used for analysis of the simulations. Electrostatic potential of the HP1α peptide and IMPα alone or the complex was calculated via the APBS sever.^72^ Molecular visualizations were generated in VMD^73^ version 1.9.3. and PyMol^54^ version 1.8.7.

## 3 RESULTS

### 3.1 Building a three‐dimensional model for better understanding structural and dynamic properties of the HP1α‐IMPα complex

**Figure 1:**
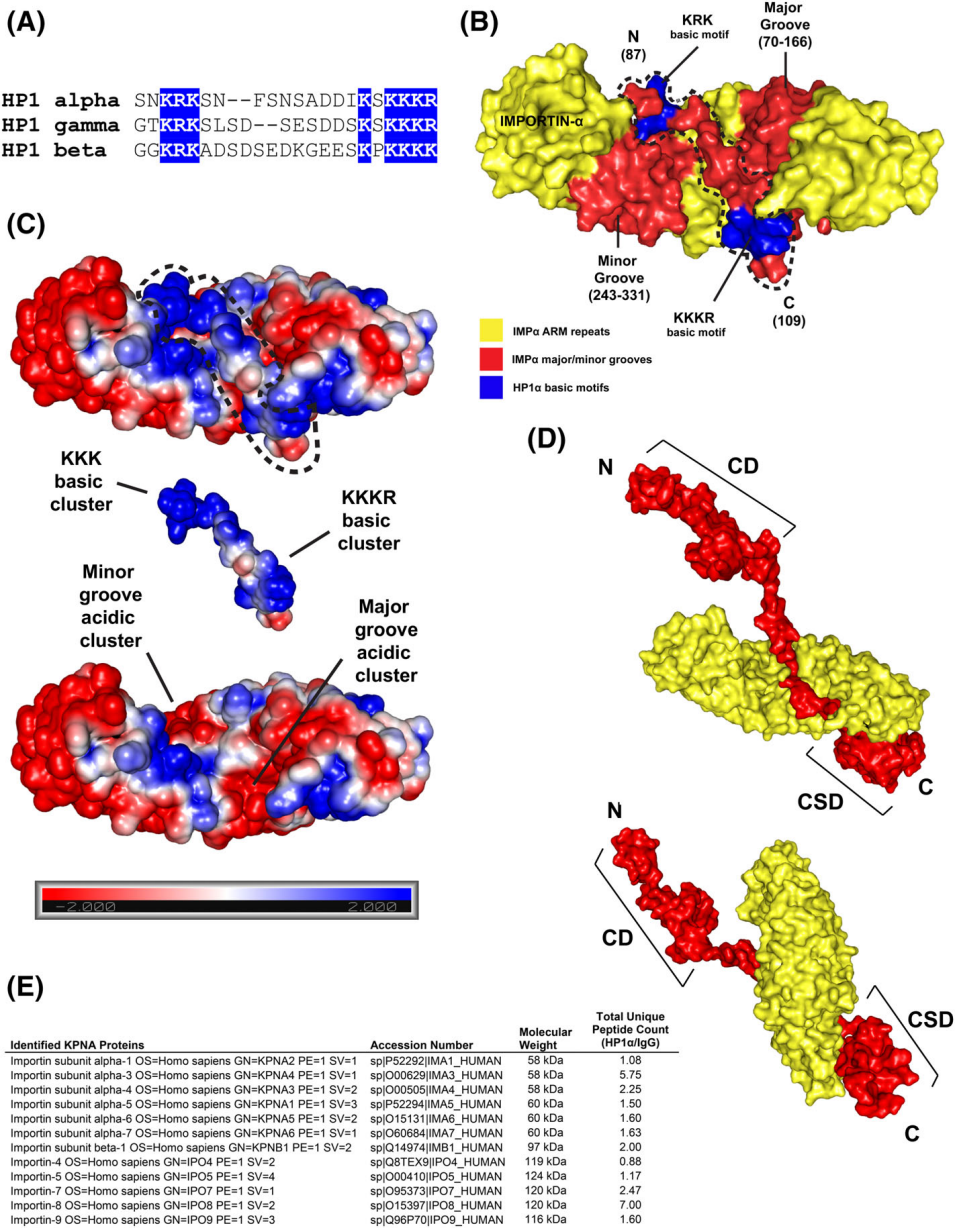
HP1α forms a complex with the nuclear receptor IMPα via the canonical binding mode. The complex of HP1α and IMPα was generated via threading the sequence of IDR2 onto the structure of an established IMPα interactor, N1N2, in Discovery Studio v17. A, Linear motif analysis of HP1α via the PsortII algorithm predicted a bipartite NLS within IDR2 that conforms to the K/RX10–12KRRK consensus sequence. This NLS is shared among the three HP1 proteins. B, 3D space‐filling model of the HP1α IDR2 peptide bound to IMPα. The N‐terminal cluster of basic residues (KRK, shown in blue) associates with the minor groove of IMPα, while the C‐terminal cluster (KKKR, shown in blue) binds to the major groove. C, Electrostatic potential of the complex, the HP1α peptide, and the IMPα receptor illustrates the charge distribution that facilitates the interaction of the HP1α IDR2 and IMPα. D, 3D space‐filling model of full‐length HP1α docked to IMPα via ClusPro. The interaction is identical to that of the IDR2 peptide with the globular CD and CSD pointing outward and downward. E, HP1α copurifies with several IMPα subunits and with IMPβ. IMPα, importin α; NLS, nuclear localization signal

The translocation of HP1α to the nucleus is a critical step for realizing its functional role in epigenetic inheritance of cancer; however, mechanisms that can regulate this critical process remained poorly studied. Notably, SLiM analysis identified a bipartite NLS within the HP1α linker (IDR2) (Figure 1A).^34^ As rules governing the binding mode of IMPα with NLS motif‐containing proteins has been previously shown,^55^ this data allowed us to develop a knowledge‐based approach to acquiring a model for the complex of HP1α and IMPα. We took advantage of threading the HP1α linker sequence onto the structure of NIN2 complexed with IMPα. In this conformation, the N‐terminal basic cluster (KRK) of the HP1α NLS associated with the minor groove of IMPα, while the C‐terminal cluster (KKKR) interacted with the major groove (Figure 1B). The electrostatic map of the complex illustrates the charge distribution of both molecules that allows HP1α to sit in the two binding pockets formed by the grooves in IMPα (Figure 1C). The complex of the full length HP1α with IMPα was also constructed through docking with ClusPro.^56^, ^57^, ^59^, ^60^ This second approach placed the NLS sequences at the same position as our threading model (Figure 1D). The interaction of HP1α and IMPα was provided by immunoaffinity purification of HP1α‐interacting proteins from HeLa cells and their mass spectrometry‐based identification. We found that HP1α copurified with several IMPα subunits and with IMPβ (Figure 1E). Thus, experimental evidence supports an interaction between these proteins, and our molecular modeling provides molecular details underlying this interaction. Last, because of the high levels of homology considered in building our models, the conclusion derived from studying their behavior has a considerable level of reliability.

### 3.2 Modeling the impact of phosphorylation on the regulation of the HP1α‐IMPα complex

**Figure 2:**
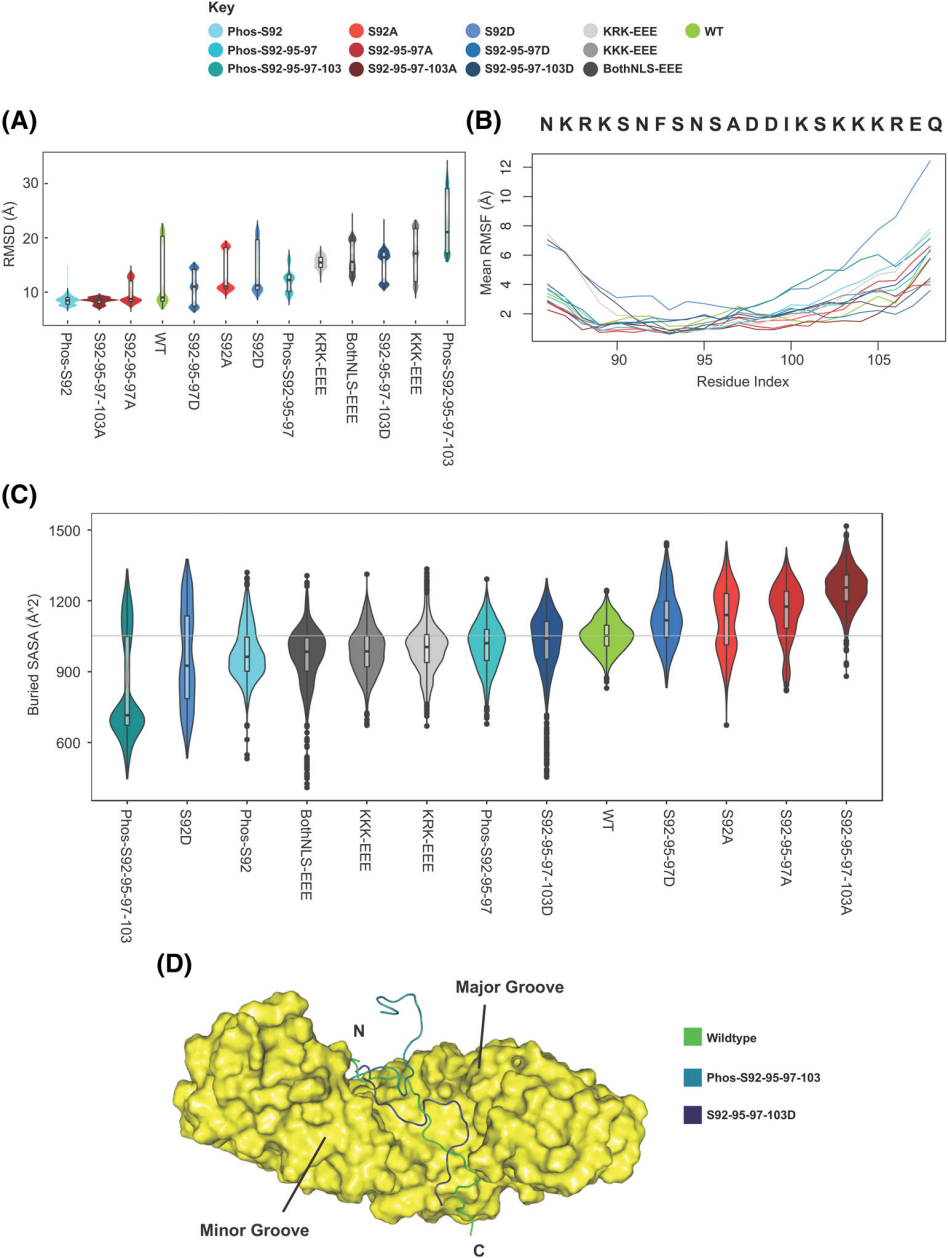
Phosphorylation of the HP1α linker inhibits binding to IMPα by affecting conformational changes in IDR2. MD simulation of the HP1α‐IMPα complex was employed to interrogate the impact of phosphorylation of the serine residues located in the region of the HP1α linker that comprises the bipartite NLS. A, B, The flexibility and mobility of the peptides in all the experimental conditions were reflected in calculations of RMSD and RMSF. The clusters of basic residues in the NLS are indicated above the RMSD plot in blue. C, Buried SASA of the HP1α peptide in all conditions. Decreased values indicate increased access to solvent. D, Visualization of the last frame of the wild‐type, Phos‐S92‐95‐97‐103, and S92‐95‐97‐103D phosphomimetic mutant trajectories was congruent with the results of RMSD and RMSF calculation and demonstrates the conformational changes sustained by the peptides. IMPα, importin α; MD, molecular dynamics; NLS, nuclear localization signal; RMSD, root‐mean‐square deviation; RMSF, root‐mean‐square‐fluctuation; SASA, solvent accessibility surface area

We and others have previously demonstrated that the linker domain of HP1α is amenable to extensive post‐translational modifications.^74^, ^75^, ^76^, ^77^ However, how these phosphorylation events affect critical functions of this protein remains to be fully understood. Thus, we considered information from SLiM analyses and data from experimentally derived mass spectrometry‐based datasets to identify phosphorylation events that most likely regulate the function of this protein. For this purpose, we generated several models of the HP1α linker including partial or fully phosphorylated linkers, as well as nonphosphorylatable, or phosphomimetic mutants in a complex with IMPα and performed MD simulations. Subsequently, we evaluated MD simulations using multiple metrics. RMSD and RMSF of each HP1α peptide quantified the overall deviation from the canonical interaction pose and which residues experienced the greatest deviations, respectively. RMSD values were increased above the range observed in the wild‐type for mutations of the NLS motifs and by phosphorylation or phosphomimetic mutation of all four serine residues in the HP1α linker peptide. Thus, these modifications likely induce changes in the linker peptide‐protein interaction (Figure 2A). RMSF (Figure 2B) showed the greatest changes in the first seven residues—specifically for S92D, N‐terminal NLS variants, and the joint NLS variants. From the middle of the peptide to the C‐terminal end, RMSF values were higher for S92D and the phosphorylation of S92‐95‐97‐103. We also assessed SASA of the HP1α peptide in each condition. The total area of the peptide was significantly reduced only by mutation of the NLS motifs, suggesting that the peptide undergoes a conformational change that resembles folding on itself (Figure S1). Generally, buried SASA of the HP1α linker peptide was reduced with phosphorylation, phosphomimetic mutations, and NLS motif mutation. Few of the conditions were associated with significant changes in total buried SASA, but there was high variability indicating the potential for rearrangements in the interaction (Figure 2C). Visualization of the last frame of the trajectories (Figure 2D and Figure S3), which represents the final conformation of the complex, summarized these overall changes, as the loss of interaction between the NLS motif residues and IMPα basic patches is visually evident. Thus, together, these global geometric measures demonstrate that the phosphomimetic mutation and phosphorylation of the serine residues within the HP1α linker alter peptide conformation.

### 3.3 Energetic and molecular mechanic calculations further assess the conformation of The HP1α‐IMPα complex

**Figure 3:**
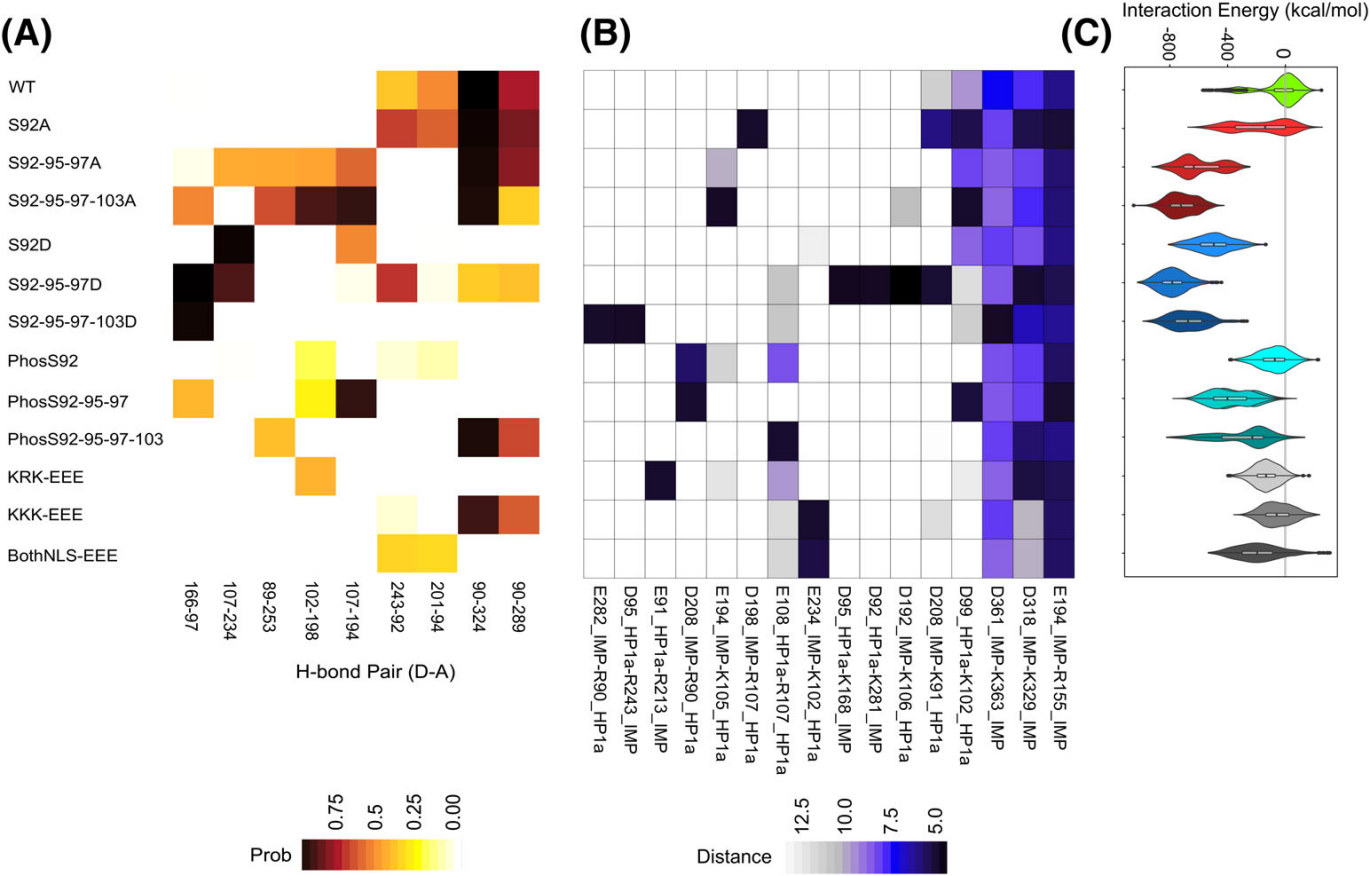
Phosphorylation of the HP1α linker prevents association with IMPα by inhibiting molecular contacts necessary for the interaction. Analysis of changes in molecular contacts throughout the MD simulations was accomplished through monitoring of noncovalent interactions and interaction energy between the two proteins. A, The hydrogen bond matrix visualizes the frequency with which each residue pair interacts, across conditions. B, The salt‐bridge matrix visualizes the median distance between donor and acceptor residue pairs. C, Interaction energy of the HP1α‐IMPα complex in each condition. The gray line represents the median value of wild‐type for comparison across conditions. IMPα, importin α; MD, molecular dynamics

We assessed changes in noncovalent bonding to infer the strength of the molecular association of the HP1α linker peptide and IMPα. The hydrogen bond matrix in Figure 3A showed that the engineered NLS binding controls, in which the basic residues in each cluster of the bipartite NLS (KRK, KKK) were mutated to acidic residues (KRK‐EEE, KKK‐EEE), produced the expected loss of hydrogen bonding and facilitated interpretation of the results of the experimental conditions. Residues R90, S92, and F94 consistently formed hydrogen bonds with residues within IMPα (eg, W201, R243, N289, and E324). Nonphosphorylatable alanine mutation of one or all the serine residues in the linker (S92, 95, 97, 103) permitted the retention of some of the same hydrogen bonds that form at the N‐terminal part of the NLS, in addition to formation of new hydrogen bonds that were not present in the wild‐type. These new interactions were between the C‐terminal basic cluster of residues (K102, R107) and residues within IMPα that are between the major and minor groves (eg, E294, D198, and E235). Thus, nonphosphorylatable variants are associated with loss of specific interactions within the major and minor groves, perhaps leading to less specific interactions.

Subsequently, we modeled the effect of serine phosphorylation of the HP1α linker domain. Phosphorylation of S92 alone, for instance, was sufficient to reduce the frequency of hydrogen bonds between HP1α and IMPα. Additional phosphorylation of S95 and S97 resulted in the formation of new hydrogen bonds, while phosphorylation of all four serine residues, S92, S95, S97, and S103 demonstrated that the peptide maintains contact with IMPα only through the hydrogen bonds formed between two basic residues in the N‐terminal portion of the NLS (K89, R90) and adjacent residues in IMPα. Monitoring salt‐bridge formation revealed that many interprotein interactions are common to all conditions, but some are variant specific (Figure 3B). Variants within the C‐terminal cluster of basic residues (KKK‐EEE) disrupted an intrachain salt‐bridge (between D318 and K320) to the minor groove of IMPα (Figure S2); specifically, K102 in HP1α to D99 in IMPα. One intrachain salt‐bridge within HP1α was compromised by the phosphorylation of S92 only, phosphorylation of S92‐95‐97‐103, mutation of one or both NLS clusters of basic residues (KKK‐EEE), and phosphomimetic mutation of S92‐95‐97 or S92‐95‐97‐103. This result indicates that the interaction between the IMPα minor grove and NLS motif residues in HP1α is crucial for the maintenance of the specific conformation of the peptide that is compatible with IMPα binding. While known biochemically, this is an important feature recapitulated in our simulations, increasing our confidence in the other results returned by our simulations. The NLS binding mutants (eg, KRK‐EEE) maintained non‐WT contact with IMPα by forming one or more salt‐bridges between one of the mutated residues and a residue outside of the IMPα major or minor groves (eg, K91E in HP1α to R213 in IMPα).

The nonphosphorylatable variants (S92‐95‐97‐103 to alanine) formed new salt bridges through K105 and K107 that contributed to the stabilization of the interaction with IMPα. Phosphorylation at S92 only or S92‐95‐97 resulted in the formation of a salt‐bridge between R90 (N‐terminal) in HP1α and D208 (major groove) in IMPα, which is congruent with the occurrence of a conformational change that causes the N‐terminal part of the HP1α NLS (KRK) to lose contact with the minor groove and make new contacts with the major groove to preserve the interaction. Only one new salt‐bridge was formed upon phosphorylation of S92‐95‐97‐103, and this intrachain interaction occurred at the end of the peptide between E108 and R107. The phosphomimetic mutants again displayed different patterns of interactions from that of the phosphorylated protein. New salt‐bridges were formed between the mutated residues (S92, S95) and nearby residues in IMPα suggesting a closer affinity of the two proteins. This observation is important as experimentalists typically consider phosphomimetic mutants to be similar to a phosphorylation event, but our data revealed differences.

Next, we computed the total interaction energy between each HP1α peptide and IMPα. The interaction energy is influenced by all other interactions quantified above, and thus the combination of metrics is more interpretable than each alone. The wild‐type and nonphosphorylatable variants showed lower interaction energies (on average, ∼−100 kcal/mol), compared to phosphorylated HP1α peptides and phosphomimetic mutants (on average, ∼−600 kcal/mol) (Figure 3C). The similarities among, for example, phosphorylations and phosphomimetic mutants make our previous observations of rearranged interprotein interaction critical for interpretation. Phosphorylation of the HP1α peptide at any site, but primarily S92, increased interaction energy similar to the NLS binding mutants, indicating that the complex is destabilized and supporting conformational changes observed in these conditions (Figure S2). Therefore, phosphorylation of the HP1α linker changes the binding mechanics with IMPα by modifying the noncovalent hydrogen bonds, salt‐bridge connections, and energy of interaction to inhibit the full interaction of the peptide with IMPα. Genomic variants lead to rearrangements of intermolecular contacts; many interactions seen in the wild type are lost for each variant and new contacts not observed in the wild type are gained (Figure S3). Because genomic variants lead to loss of interaction at the IMPα minor grove and new interaction between the IMPα groves, our data indicate that genomic variants likely alter the interaction to have less specificity.

**Figure 4:**
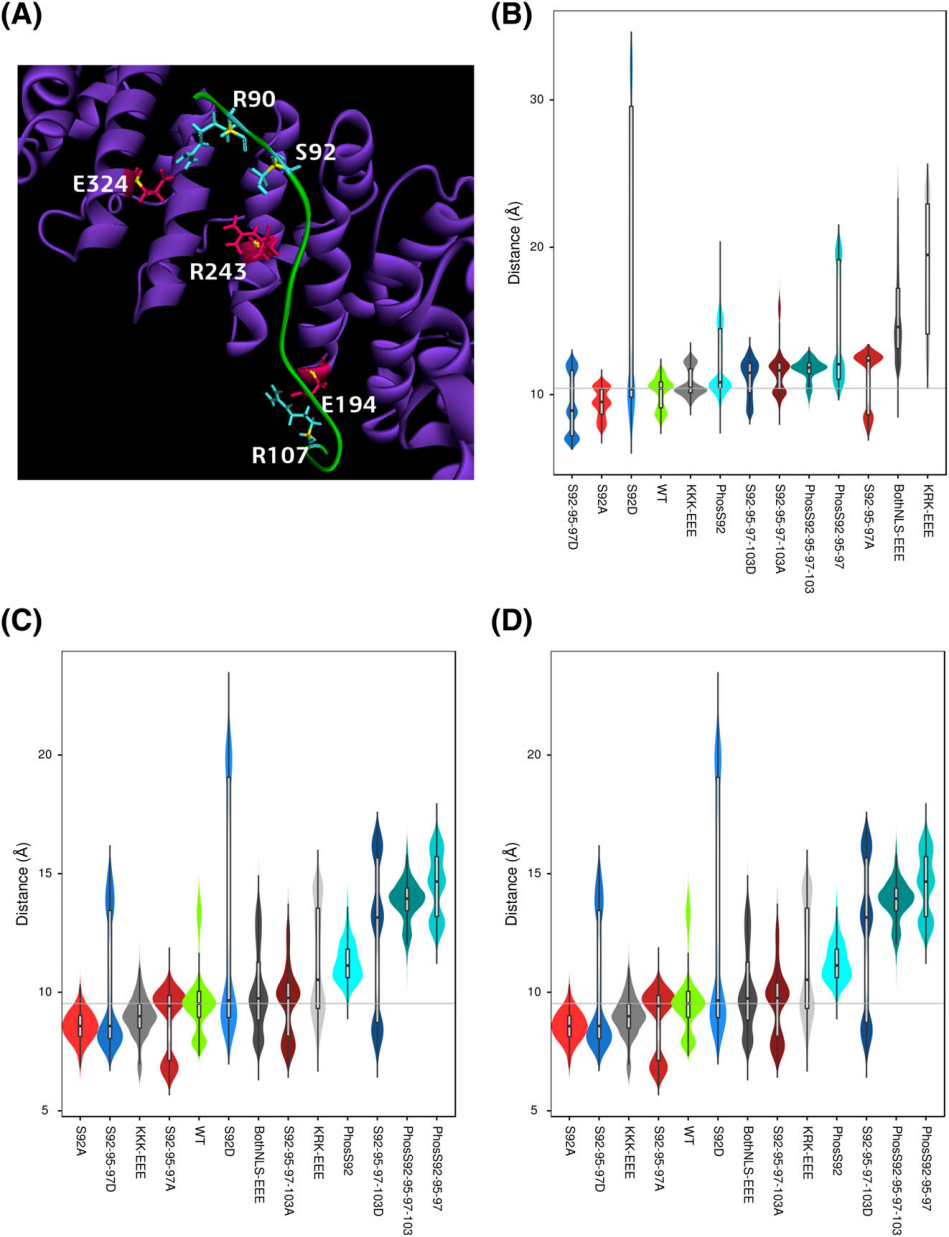
Phosphorylation of the HP1α linker increases distance between the peptide and IMPα. A, Schematic of the C^α^ atom pairs selected from the hydrogen bond matrix for receptor‐ligand distance metrics. IMPα is shown in purple with the selected residues represented as stick structures colored pink. The HP1α peptide is shown in green with the selected residues illustrated as stick structures colored cyan. All C^α^ atoms are highlighted in yellow. Residue labels are shown in white. Residue pairs for distance measurements are written with the IMPα residue appearing first. B, Distance metrics for E324:R90. C, Distance metrics for R243:S92. D, Distance metrics for E194:R107. IMPα, importin α

We also monitored the distance between residues participating in hydrogen bonds as a simplified geometric measure to facilitate interpretation (Figure 4A). The first residue pair analyzed was R90 (HP1α) and E324 (IMPα) which forms a bond in the wild‐type condition, between the N‐terminal cluster of basic residues in the HP1α NLS (KRK) and the minor groove of IMPα. The violin plot in Figure 4B shows the median value of the distance between these two residues in the wild‐type peptide, represented by a gray line. The S92D phosphomimetic mutant displayed much variability, which is attributable to the peptide shifting down and slightly out of the binding pocket created by the minor groove of IMPα (Figure S2). All phosphomimetic and nonphosphorylatable mutations impacted the conformation of the nearest NLS motif residues, often leading to the peptide leaving the major or minor grove binding site and visually evident in Figure S2 but were also associated with similar alteration at the more distant motif. The greatest separation between IMPα and HP1α was observed in the mutation of the N‐terminal cluster of basic residues to acidic ones (KRK‐EEE), because the peptide loses contact with IMPα on that end. Phosphorylation of one residue in the linker, phosphorylation of all serine residues, and for phosphomimetic substitution considerably increased the distance between S92 and R243, indicating that the post‐translational modification of the serine residues alters interactions with IMPα. The third residue pair, R107 (HP1α) and E194 (IMPα), which connect the C‐terminal part of the HP1α NLS (KKK) to the major groove of IMPα, were not found to be constantly connected by a hydrogen bond in the wild‐type simulations but were consistently present in the nonphosphorylatable mutants (Figure 3A). As an approximate quantification of how much HP1α had left the IMPα grove, we used distance monitors between specific pairs of residues that interact in the wild‐type (Figure 4C,D and S4). Distance monitors demonstrate that the peptide is dynamic within the IMPα binding site, but that consistent changes in the conformation are seen between conditions. Thus, our data indicate that phosphorylation or phosphomimetic genomic variants prevent the HP1α linker to bind IMPα.

### 3.4 Modeling the impact of phosphorylation‐specific mutations on the regulation of the HP1α‐IMPα complex

**Figure 5:**
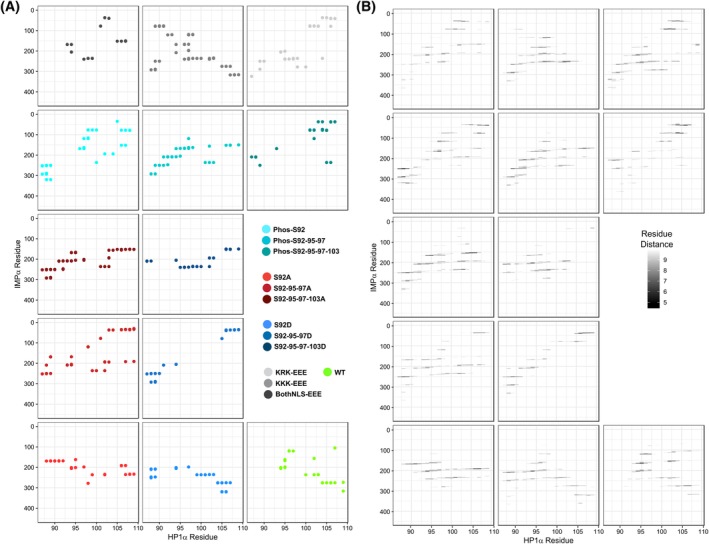
Intermolecular contacts between HP1α and IMPα in all conditions—HP1α‐IMPα intermolecular contacts are disrupted by full phosphorylation of the linker. A, Residue‐residue contacts between HP1α and IMPα were calculated and plotted in a contact map matrix where points indicate residues within 6 Å for at least 10% of the trajectory. Each condition is indicated by color, as in previous figures. B, We also plot the minimum average distance between residues on a grayscale where residues further than 10 Å apart are white and those closer than 5 Å are black. IMPα, importin α

We considered the specific patterns of residue‐level rearrangements across conditions. First, we visualized the final conformation between the two members of the complex, as a visual guide to how the interactions changed during simulation (Figure S2). We quantified rearrangements by plotting the minimum time‐averaged distance between each residue of the two proteins (Figure 5). Compared to WT, nonphosphorylatable linker variants retained some previously established contacts, but also introduced new ones, primarily with the N‐terminus. Phosphomimetic and nonphosphorylatable mutants lost more native contacts with the peptide C‐terminus and linker and gained even more interactions across the peptide N‐terminus. Interestingly, both phosphorylation and the nonphosphorylatable mutation of S92 (S92A) closely resembled WT in its bonding pattern, while its phosphomimetic mutation (S92D) induced loss of interaction between the N‐terminal portion of the HP1α peptide (residues 87‐95) and the minor groove (residues 315‐403) of IMPα (Figure 5). This finding suggests that the single phosphomimetic mutant affects changes in noncovalent interactions that secure the association of the N‐terminal end of HP1α linker to IMPα, allowing it to shift out of the binding pocket. Phosphorylation of the same residues resulted in complete loss of those C‐terminal contacts that is reflective in the last frame of the simulation (Figure S2). These data indicate a progression, where partially modified HP1α linkers retain a subset of their specific native contacts, while full phosphorylation further perturbs native contacts, resulting in the spatial separation of the molecules.

**Figure 6:**
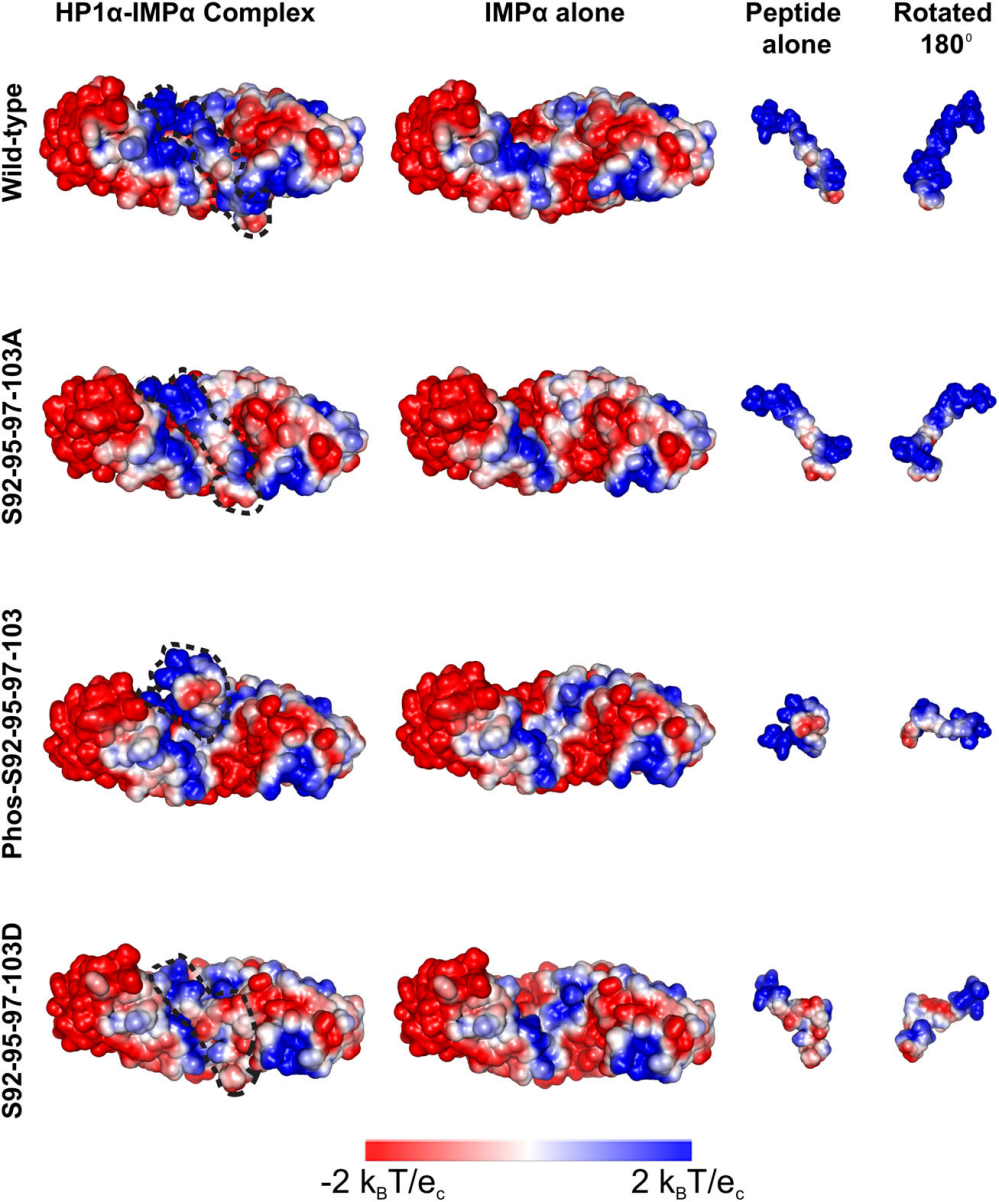
Electrostatic potential maps of the HP1α‐IMPα complexes. The charge distribution of the IMPα receptor and HP1α peptides under conditions of phosphorylation, nonphosphorylatable, and phosphomimetic mutation was evaluated and visually represented. The complex (HP1α peptide outlined in dashed lines), IMPα alone, and the peptide alone are shown with the peptide optimally rotated (180°) to expose the binding surface. IMPα, importin α

Subsequently, we analyzed conformational dynamics of the bound linker peptide using PCA. Comparing the mean values for each trajectory in PC1‐PC2 space shows variation among technical replicates (Figure S5), but with consistent trends among conditions. Phosphorylation of S92‐95‐97‐103 resulted in the most deviation from the wild‐type, while conditions with fewer phosphorylated residues and nonphosphorylatable mutants clustered together and with the wild‐type. Similar to the fully phosphorylated linker, the NLS binding mutants are separated from wild‐type in PC space, indicating their consistent global differences from the wild‐type. PC3 demonstrated relatively little consistent difference among phosphorylation or phosphomimetic mutation and wild‐type (Figure S5). However, PC2 and PC3 together showed phosphorylation of S92‐95‐97‐103 diverged from all the conditions except the NLS binding mutants (Figure S5). We also assessed changes in the charge distribution of the HP1α linker peptide upon phosphorylation, nonphosphorylatable or phosphomimetic mutation. The complex of the wild‐type HP1α peptide and IMPα demonstrated the normal conformation of the two proteins where the major and minor grove surfaces of IMPα are negatively charged to complement the positively charged peptide (Figure 6). Regions of IMPα that flank the major and minor groves are positively charged, likely helping to orient the peptide into the two groves. Therefore, we conclude that full phosphorylation of the HP1α linker is most likely to be incompatible with its transport through the nuclear membrane. This finding is congruent with the fact that the highest level of linker phosphorylation occurs during mitosis at a moment in which the nuclear membrane is not present, a phenomenon that would leave most of this form of HP1α in the cytoplasm.

### 3.5 Modeling the impact of cancer‐associated mutations on the regulation of the HP1α‐IMPα complex

To gain more insights into how the regulation of HP1α, through its linker domain, may be altered by cancer‐associated mutations, we evaluated six additional genomic variants, applying the same approaches used above to assess phosphorylation. Peptide RMSD measures global changes from the initial contacts; S92Y and Q109H were associated with smaller deviations, while F94L and D99A were associated with larger departures (Figure S6A). RMSF values indicated that the C‐terminal side is more mobile than the N‐terminal side for WT and the majority of variants. This mobility increased for F94L and D99A compared to all other conditions (Figure S6B). Buried SASA was increased for S92Y and S95L but decreased for F94L (Figure S6C). The total SASA of the peptide was increased for S92Y and decreased for F94L (Figure S6D), indicating rearrangements to intrapeptide interactions. Visualization of the final conformation for each simulation demonstrated differences in positioning of the center of the peptide, which was no longer between the IMPα groves for many variants. Lastly, the C‐terminus often departed from the major grove acidic cluster (Figure S7).

We next analyzed interacting residues at the binding interface between both proteins. Certain hydrogen bonds between the peptide and IMPα were stable across all variants. For example, the hydrogen bond between R90 in HP1α and E324 in IMPα was stable in all but D99A. However, all the variants formed new bonds with IMPα (S4A). Interestingly, the D99A and I101M variants, which occurred relatively close to the C‐terminal end of the HP1α linker peptide, formed additional hydrogen bonds through residues K91 and S92 at the N‐terminal end of the peptide. Similar to wild‐type, I101M did not establish any hydrogen bond contacts with the C‐terminal end of the peptide. Analysis of salt‐bridge interactions showed that one HP1α intrachain salt‐bridge (D99 and K102) was abolished by the D99A and S95L variants; with D99A also forming a new interchain salt‐bridge with R166 in IMPα (Figure S4B). The F94L variant formed two new salt‐bridges (D100 in HP1α and R155 in IMPα; K102 in HP1α and D198 in IMPα) that could further stabilize binding of the C‐terminal end of the peptide to the major groove of IMPα. The total interaction energy was lower for F94L and S95L and higher for the S92Y variant (Figure S4C), indicating tighter and weaker interaction, respectively. We assessed residue‐residue proximity for all pairs, which revealed an increase in the number of intermolecular contacts formed between the HP1α variants and IMPα, which clearly indicated the extent of rearranged interactions for the majority of variants (Figure S5). Normally, the HP1α linker peptide makes specific contacts with the IMPα major and minor groves. Thus, altogether, the interaction metrics between HP1α and IMPα suggest that certain genomic variants rearrange intermolecular contacts in a manner to render HP1α less specific for the IMPα major and minor groves, likely leading to impaired function.

## 4 DISCUSSION

Our laboratory has studied HP1 proteins for several years in relationship to their function as epigenomic effectors of growth signaling pathways. Interestingly, our own work and that of others has been, throughout the years, biased to studying the structural‐functional relationship of the globular domain of these proteins. Thus, the current study, which is focused on using molecular modeling and dynamic stimulations to interpret HP1α linker phosphorylation and cancer‐associated genomic variants bears novelty and biomedical relevance. In particular, we specifically investigate how these regulatory and pathological events alter interactions with the nuclear receptor, IMPα. Linear motif analyses identify, with a high level of confidence, an NLS consensus site within this intrinsically disordered region of the protein. This data is congruent with our immunopurification experiments that reveal the existence of a HP1α‐IMPα complex. Linear Motif analyses for post‐translational modifications combined with data from large scale mass spectrometry experiments demonstrate that the linker is amenable to phosphorylation at different sites. Thus, the HP1α linker appears to function as a binding interface for IMPα and in the integration for upstream regulatory cell signaling pathways. More importantly, this complex is predicted to be responsible of transporting HP1α into the nucleus, where it becomes functional. We find that several cancer‐associated mutations affect the sequence of this domain in a manner that we predicted may disrupt these functions. Indeed, molecular modeling and dynamic simulations, combined with an analytical approach that uses different metrics, provide evidence to support this prediction. Briefly, we find that the unmodified linker peptide binds to IMPα via hydrogen bonds and salt‐bridges formed between the bipartite NLS of HP1α and acidic patches within the major and minor grooves of IMPα. The selected variants studied here were rare missense mutations reported in various cancers but not in the general population. Our modeling experiments show that the interaction between these proteins is inhibited by phosphorylation of the four serine residues within the linker, which leads to a more extended HP1α conformation that is not compatible with the canonical binding mode for IMPα. Missense mutations in the HP1α linker rearranged interactions with IMPα, likely leading to loss of binding or nonspecific binding. The changes in linker conformation were mediated by modification of intramolecular and intermolecular contacts that reflect the dynamic property of the linker peptide. Interestingly, phosphorylation of S92 might be sufficient to disrupt the association of the HP1α linker with IMPα; however, the phosphomimetic mutant of the same residue was more potent at shifting the peptide out of the IMPα binding pocket. Furthermore, the phosphorylation of S92 only or even phosphorylation of S92‐95‐S97 did not achieve the full effect in the form of reduced binding affinity for IMPα, as did phosphorylation of all four serine residues (S92, S95, S97, S103), suggesting that all four residues may affect the mechanism of IMPα interaction. These data support our own and previous findings that each serine residue within the NLS is phosphorylated during mitosis.^74^, ^75^, ^76^ The S92Y and S95L variants could potentially disrupt the aforementioned phosphorylation‐dephosphorylation cycle and present problems for accurately completing mitosis and nuclear translocation of HP1α. The loss of interaction at the N‐terminus potentially modifies the flexibility of the peptide, allowing the new interactions to occur. Our results demonstrate the dynamics of hydrogen bond chemistry that govern the interaction between IMPα and the unmodified or phosphorylated HP1α linker. Hence, this new knowledge extends our understanding of the functional impact that both, signaling and cancer‐associated modifications may have on HP1α‐IMPα interactions. Thus, this information, generated at an atomic resolution level, should help to draw important inferences that explain critical functions of this key epigenetic regulator. In fact, one may consider that HP1α binding to IMPα via its linker NLS is of paramount functional importance. The formation of an HP1α‐IMPα complex is a necessary step for subsequent translocation of the former through the nuclear membrane into the nucleoplasm; and ultimately to chromatin, where it exercises gene regulatory functions. The current study predicts that hyperphosphorylation of the linker domain of this protein likely prevents its association with IMPα. This information is congruent with studies that show that this phenomenon takes place during mitosis, the time in which the nuclear membrane has dissolved. However, insights into how these post‐translational modifications achieve these effects, at an atomic resolution level, had remained unknown until this study. An additional contribution of this study is the modeling of cancer‐associated HP1α mutants within the linker domain of this protein, which also predict to disrupt interactions in tumor samples. Notably, this mechanism of dysfunction has never been explored before for HP1α. We would also like to briefly consider that the current study can serve as a model for understanding the interaction of IMPα to other proteins, for which the NLS is similar in content and distribution of acceptor motifs for phosphorylation. Similarly, changes in the surface of the HP1α linker under phosphorylated or dephosphorylated conditions may allow predictions for its interaction with other proteins or DNA. Consequently, these are few examples of how our results not only offer explanations of mechanisms underlying functional aspects of these proteins, but also have predictive value, such as for understanding how somatic mutations in the linker region may lead to functional inactivation in nonmitotic cells.

## CONFLICT OF INTEREST

The authors declare no potential conflict of interest.

## Supporting information


**Table S1. Multiple Sequence Alignment (MSA) of human HP1α, HP1γ, and HP1β.** The MSA was used for comparison of shared sequence and structure among the HP1 isoforms. Protein sequences were acquired from UniProt. Reported SNVs in disease and normal populations are shown in the first row. MESSA predictions of secondary structures (alpha‐Helix; beta‐shEet; coils) and disorder predictions (shown by asterisks highlighted in gray) are shown in the second and third rows, respectively.
**Table S2 HP1α is phosphorylated at serine residues within the linker by numerous kinases**.
**Table S3 Genomic variants reported in HP1α (*CBX5*)**. COSMIC and TCGA account for mutations observed in various cancers, while GnomAD register disease‐ specific variants and variants identified in population genetic studies.
**Table S4 Genomic variants identified in the HP1α linker and their pathogenicity predictions.** For the current study, the variants assessed were concentrated in the linker/IDR2 region. All variants found for this region in the gnomAD browser were excluded since they were present in phenotypically normal individuals. The degree of pathogenicity of each variant was calculated using several in silico predictive algorithms recommended by the ACMG and the results tabulated. Unfortunately, the correlation between each platform was low; therefore, the variants were considered VUS for this study.
**Table S5 Genomic variants and experimental mutations of HP1α utilized in this study**. We catalog genomic variants reported in the HP1α linker and engineered variants made for the purpose of analyzing the role of phosphorylation in regulating the interaction of HP1α with IMPα.
**Figure S1 Phosphorylation of the HP1α linker reduces the buried SASA of the peptide. (A)** Total surface area of the HP1α linker peptide is reduced only with mutation of the basic residues in the NLS binding motifs (KRK or KKK or both) to acidic residues.
**Figure S2 Visualization of superimposed final conformations of the HP1α linker peptide bound to IMPα in related conditions.** Conditions are grouped based on the residue (s) of interest in comparison to wild‐type. **(A)** S92 conditions **(B)** S92‐95‐97 conditions **(C)** S92‐95‐97‐103 conditions **(D)** NLS binding mutant conditions.
**Figure S3 Genomic variants strengthen and increase the number of Intermolecular contacts between HP1α and IMPα.** Residue‐residue contacts between HP1α and IMPα were calculated and plotted in a contact map matrix. **A)** Points indicate residue pairs that are within 6 Å. **B)** A grayscale gradient indicates average proximity.
**Figure S4 Genomic variants in the HP1α linker promote reorganizing of the intermolecular contacts to stabilize the complex with IMPα. (A)** Hydrogen bond matrix of hydrogen bond donor and acceptor pairs in all conditions. The constant presence (probability ≥0.5) or absence (probability ≤0.5) of a hydrogen bond is defined by the black to white color scale. **(B)** The salt‐bridge matrix uses the distribution value of 7.5 as a cut‐off for the presence of a salt‐bridge between the donor and acceptor pair. Colors in the range of light purple to black indicate the presence of a salt‐bridge. Chain A refers to IMPα and chain B represents the HP1α peptide. **(C)** Total interaction energy of the HP1α‐IMPα complex across all conditions. Negative kcal/mol values represent the energy of unbinding and destabilization of the complex. Values closer to or greater than zero indicate a stable interaction. **(D‐F)** Distance metrics of each residue pair as outlined in Figure 4(C).
**Figure S5 Phosphorylation and phosphomimetic mutation of all serine residues in the HP1α linker increases deviation from the wild‐type conformation.** Principle component analysis of all frames of the trajectories from each experimental condition demonstrates an overall divergence of the fully phosphorylated or phosphomimetic HP1α peptide from wild‐type and all other conditions. **(A)** Comparison of PC1 and PC2 shows clustering of the wild‐type, non‐phosphorylatable, and partially phosphorylated or phosphomimetic peptides. **(B)** Comparison of PC1 and PC3 illustrates the similarity via clustering together of the phosphorylation or phosphomimetic mutation of S92 through S103. **(C)** Comparison of PC2 and PC3 shows that phosphorylation of S92 through S103 diverged from all the conditions except the NLS binding mutants. **HP1α linker genomic variants are comparable to the wild‐type protein.** Principle component analysis of the trajectories demonstrates clustering of the variants with wild‐type with the exception of two replicate outliers from wild‐type and the I101M variant. **(D)** PC1 vs PC2 **(E)** PC1 vs PC3 **(F)** PC2 vs PC3.
**Figure S6 Genomic variants in the HP1α linker do not drastically affect the overall mobility or conformation of the peptide. (A)** RMSD calculation illustrates little deviation of the variants from wild‐type but increased variability similar to wild‐type in the I101M and Q109H variants. **(B)** RMSF calculation shows the F94 L variant diverges most from wild‐type. **(C)** Buried SASA of the HP1α linker peptide in all conditions. **(D)** Total surface area of the HP1α linker peptide in all conditions.
**Figure S7 Visualization of the final conformation of the HP1α linker variant peptides bound to IMPα. (A)** Wild‐type **(B)** S92Y **(C)** F94 L **(D)** S95 L **(E)** D99A **(F)** I101M **(G)** Q109H **(H)** Superimposition of all peptides bound to IMPα.
