# Supplementary material for: Modeling post‐translational modifications and cancer‐associated mutations that impact the heterochromatin protein 1α‐importin α heterodimers
Source: Proteins. 2019 Jun 14;87(11):904–16. doi: 10.1002/prot.25752 (PMC6790107; doi:10.1002/prot.25752)
Supplement: Supplementary file 1 — Table S1. Multiple Sequence Alignment (MSA) of human HP1α, HP1γ, and HP1β. The MSA was used for comparison of shared sequence and structure among the HP1 isoforms. Protein sequences were acquired from UniProt. Reported SNVs in disease and normal populations are shown in the first row. MESSA predictions of secondary structures (alpha‐Helix; beta‐shEet; coils) and disorder predictions (shown by asterisks highlighted in gray) are shown in the second and third rows, respectively. Table S2 HP1α is phosphorylated at serine residues within the linker by numerous kinases. Table S3 Genomic variants reported in HP1α (CBX5). COSMIC and TCGA account for mutations observed in various cancers, while GnomAD register disease‐ specific variants and variants identified in population genetic studies. Table S4 Genomic variants identified in the HP1α linker and their pathogenicity predictions. For the current study, the variants assessed were concentrated in the linker/IDR2 region. All variants found for this region in the gnomAD browser were excluded since they were present in phenotypically normal individuals. The degree of pathogenicity of each variant was calculated using several in silico predictive algorithms recommended by the ACMG and the results tabulated. Unfortunately, the correlation between each platform was low; therefore, the variants were considered VUS for this study. Table S5 Genomic variants and experimental mutations of HP1α utilized in this study. We catalog genomic variants reported in the HP1α linker and engineered variants made for the purpose of analyzing the role of phosphorylation in regulating the interaction of HP1α with IMPα. Figure S1 Phosphorylation of the HP1α linker reduces the buried SASA of the peptide. (A) Total surface area of the HP1α linker peptide is reduced only with mutation of the basic residues in the NLS binding motifs (KRK or KKK or both) to acidic residues. Figure S2 Visualization of superimposed final conformations of the HP1α linker pept [file PROT-87-904-s001.pdf]

SUPPLEMENTARY FIGURES AND TABLES

MODELING POST-TRANSLATIONAL MODIFICATIONS AND CANCER-ASSOCIATED MUTATIONS THAT IMPACT THE HETEROCHROMATIN PROTEIN 1α-IMPORTIN α HETERODIMERS

Michael T. Zimmermann[1,2], Monique M. Williams[3], Eric W. Klee[3], Gwen A. Lomberk[4,5,6,*], and Raul Urrutia[4,6,7*]

[1]Bioinformatics Research and Development Laboratory, and Precision Medicine Simulation Unit, Genomics and Precision Medicine Center (GSPMC), Medical College of Wisconsin, Milwaukee, WI

[2]Clinical and Translational Sciences Institute, Medical College of Wisconsin, Milwaukee, WI

[3]Departments of Biochemistry and Biostatistics, Mayo Clinic, Rochester, MN

[4]Division of Research, Department of Surgery, Medical College of Wisconsin, Milwaukee, WI

Center, Medical College of Wisconsin, Milwaukee, WI

[5]Department of Pharmacology and Toxicology, Medical College of Wisconsin, Milwaukee, WI

[6]Genomics and Precision Medicine Center (GSPMC), Medical College of Wisconsin, Milwaukee, WI

[7]Department of Biochemistry, Medical College of Wisconsin, Milwaukee, WI

*Corresponding authors: glomberk@mcw.edu or rurrutia@mcw.edu

Table S1: Multiple Sequence Alignment (MSA) of human HP1α, HP1γ, and HP1β. The MSA was used for comparison of shared sequence and structure among the HP1 isoforms. Protein sequences were acquired from UniProt. Reported SNVs in disease and normal populations are shown in the first row. MESSA predictions of secondary structures (alpha-Helix; beta-shEet; coils) and disorder predictions (shown by asterisks highlighted in gray) are shown in the second and third rows, respectively.

```
                               NP   N                 SH          Q       Q
HP1 alpha SNVs             RN  MQ   NN     AK  I      MCM     K   T     K R
HP1 alpha Secondary Struc ccccccccccccccccccccEEEEEEEEEEEEccEEEEEEEEcccccccc
MESSA Disorder            ********************YVVEKVLDRRVVKGQVEYLLKWKGFSEEHN
HP1 alpha_HUMAN ----------MGKKTKR-TADSSSSEDEEEYVVEKVLDRRVVKGQVEYLLKWKGFSEEHN
HP1 alpha_MOUSE ----------MGKKTKR-TADSSSSEDEEEYVVEKVLDRRMVKGQVEYLLKWKGFSEEHN
HP1 gamma_HUMAN MASNKTTLQKMGKKQNGK-SKKVEEAEPEEFVVEKVLDRRVVNGKVEYFLKWKGFTDADN
HP1 gamma_MOUSE MASNKTTLQKMGKKQNGK-SKKVEEAEPEEFVVEKVLDRRVVNGKVEYFLKWKGFTDADN
HP1 beta_HUMAN  ----------MGKKQNKKKVEEVLEEEEEEYVVEKVLDRRVVKGKVEYLLKWKGFSDEDN
HP1 beta_MOUSE  ----------MGKKQNKKKVEEVLEEEEEEYVVEKVLDRRVVKGKVEYLLKWKGFSDEDN
                          **** :    ..  . : **:*********:*:*:***:******:: .*


                                          V                        E
                           D          E   S                        N
HP1 alpha SNVs       TSF   V   Q      NL  C           S   YS  LL   A M
HP1 alpha SS    cccccccccccHHHHHHHHHHHHHHcccccccccccccHHHccccccccccccHHHHHHc
MESSA Disorder  TWEPEKNLDCPELISEF*******************************************
HP1 alpha_HUMAN TWEPEKNLDCPELISEFMKKYKKMKEGENNKPREKSESNKRKSN--FSNSADDIKSKKKR
HP1 alpha_MOUSE TWEPEKNLDCPELISEFMKKYKKMKEGENNKPREKSEGNKRKSS--FSNSADDIKSKKKR
HP1 gamma_HUMAN TWEPEENLDCPELIEAFLNSQKAGKEKD---------GTKRKSLSD--SESDDSKSKK-K
HP1 gamma_MOUSE TWEPEENLDCPELIEDFLNSQKAGKEKD---------GTKRKSLSD--SESDDSKSKK-K
HP1 beta_HUMAN  TWEPEENLDCPDLIAEFLQSQKTAHETDK------SEGGKRKADSDSEDKGEESKPKK-K
HP1 beta_MOUSE  TWEPEENLDCPDLIAEFLQSQKTAHETDK------SEGGKRKADSDSEDKGEESKPKK-K
                *****:*****:**  *::. *  :* :         . ***:     ...:: * ** :


                 E  V  L
HP1 alpha SNVs   HNST  W  G     D  T  P  WD  V      G       F       Y H  VTY
HP1 alpha SS    ccccccccccccccccccccccccccccEEEEEEEccccccHHHHHHHccccccHHHHHH
MESSA Disorder  **********************TDSCGDLMFLMKWKDTDEADLVLAKEANVKCPQIVIAF
HP1 alpha_HUMAN EQSNDIARGFERGLEPEKIIGATDSCGDLMFLMKWKDTDEADLVLAKEANVKCPQIVIAF
HP1 alpha_MOUSE EQSNDIARGFERGLEPEKIIGATDSCGDLMFLMKWKDTDEADLVLAKEANVKCPQIVIAF
```

```
HP1 gamma_HUMAN RDAADKPRGFARGLDPERIIGATDSSGELMFLMKWKDSDEADLVLAKEANMKCPQIVIAF
HP1 gamma_MOUSE RDAADKPRGFARGLDPERIIGATDSSGELMFLMKWKDSDEADLVLAKEANMKCPQIVIAF
HP1 beta_HUMAN  KEESEKPRGFARGLEPERIIGATDSSGELMFLMKWKNSDEADLVPAKEANVKCPQVVISF
HP1 beta_MOUSE  KEESEKPRGFARGLEPERIIGATDSSGELMFLMKWKNSDEADLVPAKEANVKCPQVVISF
                .:  :  *** ***:**:*******.*:********::****** *****:****:**:*

                          T V     N
HP1 alpha SNVs    D    Y HS NV S DI I  I
HP1 alpha SS     HHHHHcccccccccHHHHHHHccc
MESSA Disorder  YEERLT******************
HP1 alpha_HUMAN YEERLTWHAYPEDAENKEKETAKS       191
HP1 alpha_MOUSE YEERLTWHAYPEDAENKEKESAKS       191
HP1 gamma_HUMAN YEERLTWHSCPEDEAQ--------       183
HP1 gamma_MOUSE YEERLTWHSCPEDEAQ--------       183
HP1 beta_HUMAN  YEERLTWHSYPSEDDDKKDDKN--       185
HP1 beta_MOUSE  YEERLTWHSYPSEDDDKKDDKN--       185
                ********: *.:  :
```

Table S2: HP1α is phosphorylated at serine residues within the linker by numerous kinases.

Residue	NetPhos	KinasePhos	PHOSIDA	DISPHOS	GPS	Phosphosite Plus	Phospho SVM	Potential Kinases
11	Yes	Yes	Yes	Yes	Yes	Yes	Yes	PKB, ATM, IKK, PLK1, CKI, CK2
12	Yes	Yes	Yes	Yes	Yes	Yes	Yes	ATM, AURK, IKK, PLK1, CK1, CK2
13	Yes	Yes	Yes	Yes	Yes	Yes	Yes	ATM, IKK, CK1, CK2
14	Yes	Yes	Yes	Yes	Yes	Yes	Yes	PKB, ATM, CK2
45	Yes	Yes	Yes	-	Yes	Yes	-	ATM, PKC, CaM
64	-	Yes	-	-	Yes	-	-	PKB, ATM, PKC
85	Yes	Yes	Yes	Yes	Yes	-	Yes	PKB, AURK, ATM, IKK, PLK1
87	Yes	Yes	Yes	Yes	Yes	-	Yes	ATM, AURK, PLK1, CK1
92	Yes	Yes	Yes	Yes	Yes	Yes	Yes	PKB, ATM, PKC, AURK, PLK1, ROCK
95	Yes	Yes	Yes	Yes	Yes	Yes	Yes	PKA, ATM, PKC, AURK, PLK1, NDR
97	Yes	Yes	Yes	Yes	Yes	Yes	Yes	ATM, AURK, PKG, CK2
103	Yes	Yes	Yes	Yes	Yes	Yes	-	PKB, ATM, CDK
110	Yes	Yes	Yes	Yes	Yes	Yes	Yes	PKA, PKB, ATM, PKC, IKK, PLK1, CK1
132	Yes	Yes	Yes	-	Yes	Yes	-	ATM, CK2
191	Yes	Yes	Yes	Yes	Yes	Yes	Yes	DMPK, PEK, PKC, BARK

Potential kinases were filtered with a 0.6 cut-off value to exclude non-specific predictions.

Table S3: Genomic variants reported in HP1α (*CBX5*). COSMIC and TCGA account for mutations observed in various cancers, while GnomAD register disease- specific variants and variants identified in population genetic studies.

**Domain**	**COSMIC**	**TCGA**	**GnomAD**
**IDR1 (1-19)**	G2R, K6M, S14del	K3N	R7Q, D10N, E18K,
**Chromodomain (20-78)**	R28M, R28S, R29C, V30M, E46K, H48R, K55T, L57F, E61V, E61D, E65Q, G76C	R28S, R28M, R29C, V30M, E36K, K40T, E46K, H48R, N56S, E61D, K72N	V21I, R29H, V30M, K40Q, H48Q, M73L, G76S,
**IDR2 (79-120)**	S92Y, S95L, D99A, D99N, I101M, R115W, R115L	F94L, S95L, D99A, I101M, Q109H, R115W, E118G,	N88S, N93S, D99N, Q109E, S110N,
**Chromoshadow domain (121-179)**	T130P, M137V, L152F, C160Y, A166T, E170D, H175Y, P178S,	E124D, M137V, L152F, C160Y, Q162H	I165V, Y177H
**IDR3 (179-191)**	E185D	D180N, E185D, K186I, K186N, S191I	A181V, N183S, T188I

Table S4: Genomic variants identified in the HP1α linker and their pathogenicity predictions. For the current study, the variants assessed were concentrated in the linker/IDR2 region. All variants found for this region in the gnomAD browser were excluded since they were present in phenotypically normal individuals. The degree of pathogenicity of each variant was calculated using several *in silico* predictive algorithms recommended by the ACMG and the results tabulated. Unfortunately, the correlation between each platform was low; therefore, the variants were considered VUS for this study.

Variant	Mutation Taster Prediction and Grantham Score*	PPH2**	MutPred2***	SIFT/PROVEAN****
**S92Y, c.275C>A**	Disease-causing, 144	Benign	Benign, 0.361	Damaging
**F94L, c.282C>A**	Polymorphism, 22	Benign	Benign, 0.232	Tolerated
**S95L, c.284C>T**	Disease-causing, 145	Benign	Benign, 0.186	Tolerated
**D99A, c.296A>C**	Disease-causing, 126	Benign	Benign, 0.315	Tolerated
**I101M, c.303C>G**	Disease-causing, 10	Possibly Damaging	Benign, 0.15	Tolerated
**Q109H, c.327G>T**	Disease-causing, 24	Possibly Damaging	Benign, 0.335	Tolerated

** The Grantham score represents the divergence of the amino acids. A higher score indicates a greater difference between the primary residue and the substituted one and signifies potential change in biochemistry at that position.

** PolyPhen2 predicts possible influence of amino acid substitution on the overall structure and function of the protein.

*** MutPred2 calculates the impact of amino acid substitution on protein structure and mechanism of action based on 50 different protein characteristics. A cut-off score of 0.611 designates a variant as pathogenic [66].

**** SIFT/PROVEAN predicts the impact of amino acid substitution on the biological function of the protein [67].

Table S5: Genomic variants and experimental mutations of HP1α utilized in this study. We catalog genomic variants reported in the HP1α linker and engineered variants made for the purpose of analyzing the role of phosphorylation in regulating the interaction of HP1α with IMPα.

Observed Variant	Engineered Variant and Post-Translational Mark	Experimental Mutation Classification
S92Y, c.275C>A	Phos-S92	Phosphorylated
F94L, c.282C>A	Phos-S92-95-97	
S95L, c.284C>T	Phos-S92-95-97-103	
D99A, c.296A>C	S92A	Non-phosphorylatable
I101M, c.303C>G	S92-95-97A	
Q109H, c.327G>T	S92-95-97-103A	
	S92D	Phosphomimetic
	S92-95-97D	
	S92-95-97-103D	
	KRK-EEE (N-terminal)	Unbinding
	KKK-EEE (C-terminal)	
	BothNLS-EEE	

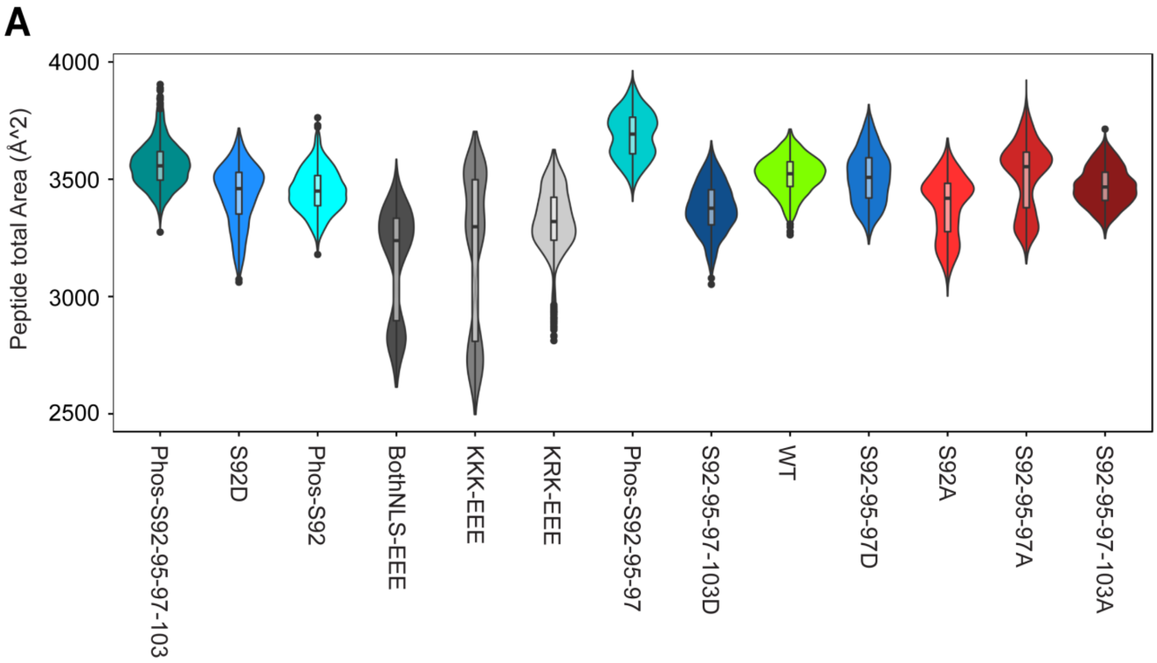

Figure S1: Phosphorylation of the HP1α linker reduces the buried SASA of the peptide. (A) Total surface area of the HP1α linker peptide is reduced only with mutation of the basic residues in the NLS binding motifs (KRK or KKK or both) to acidic residues.

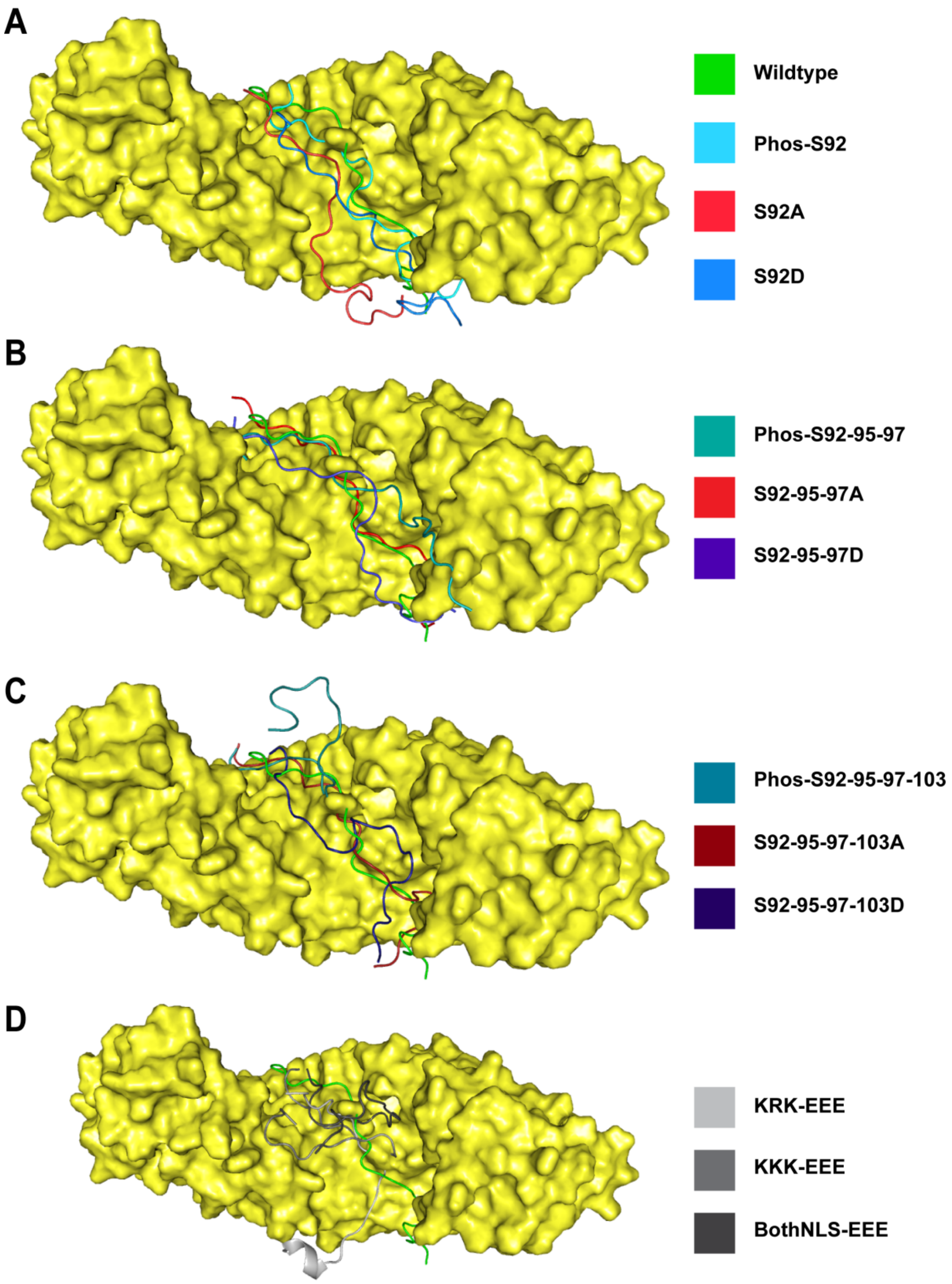

Figure S2: Visualization of superimposed final conformations of the HP1α linker peptide bound to IMPα in related conditions. Conditions are grouped based on the residue (s) of interest in comparison to wild-type. **(A)** S92 conditions **(B)** S92-95-97 conditions **(C)** S92-95-97-103 conditions **(D)** NLS binding mutant conditions.

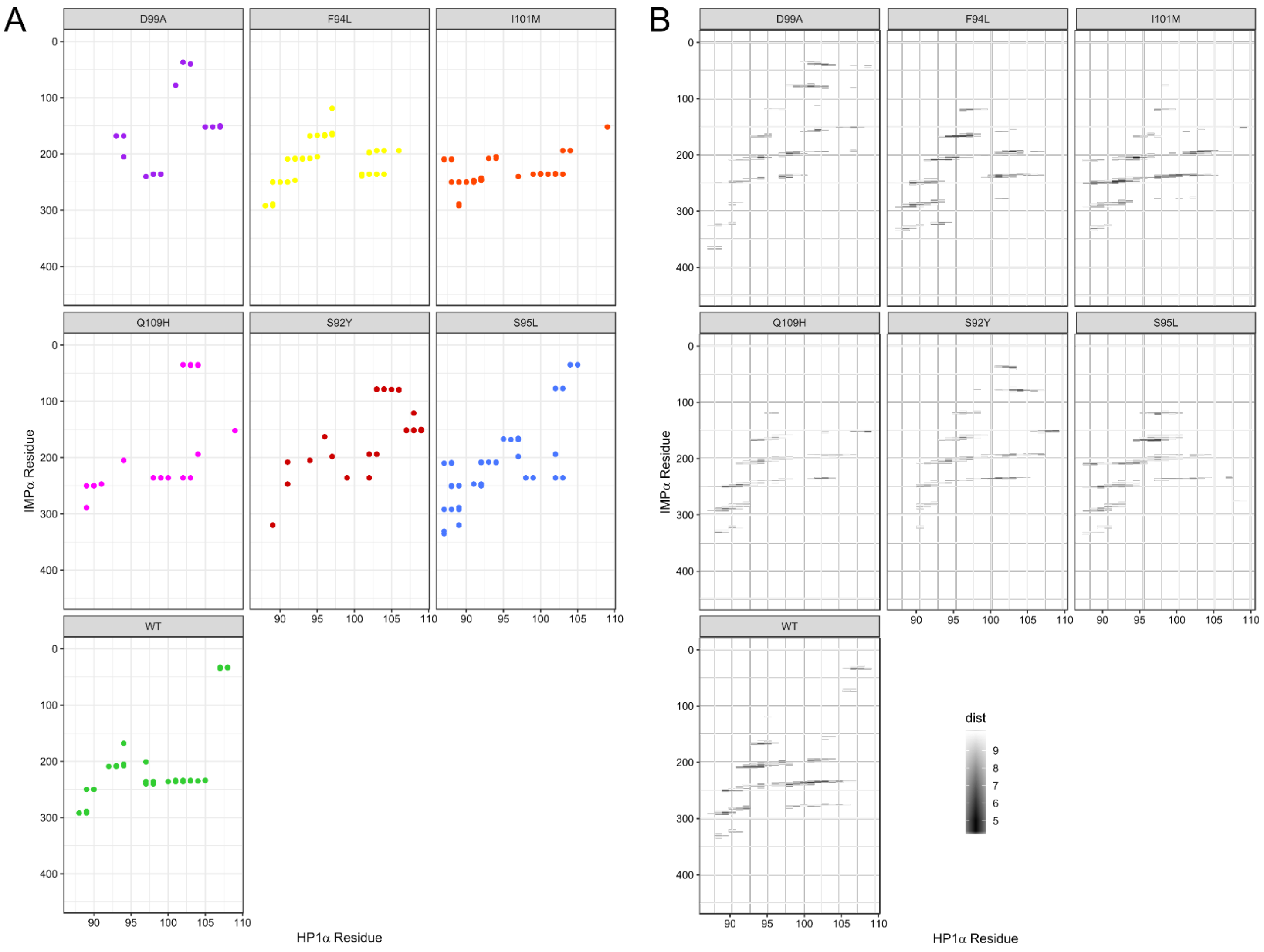

Figure S3: Genomic variants strengthen and increase the number of Intermolecular contacts between HP1α and IMPα. Residue-residue contacts between HP1α and IMPα were calculated and plotted in a contact map matrix. **A)** Points indicate residue pairs that are within 6Å. **B)** A grayscale gradient indicates average proximity.

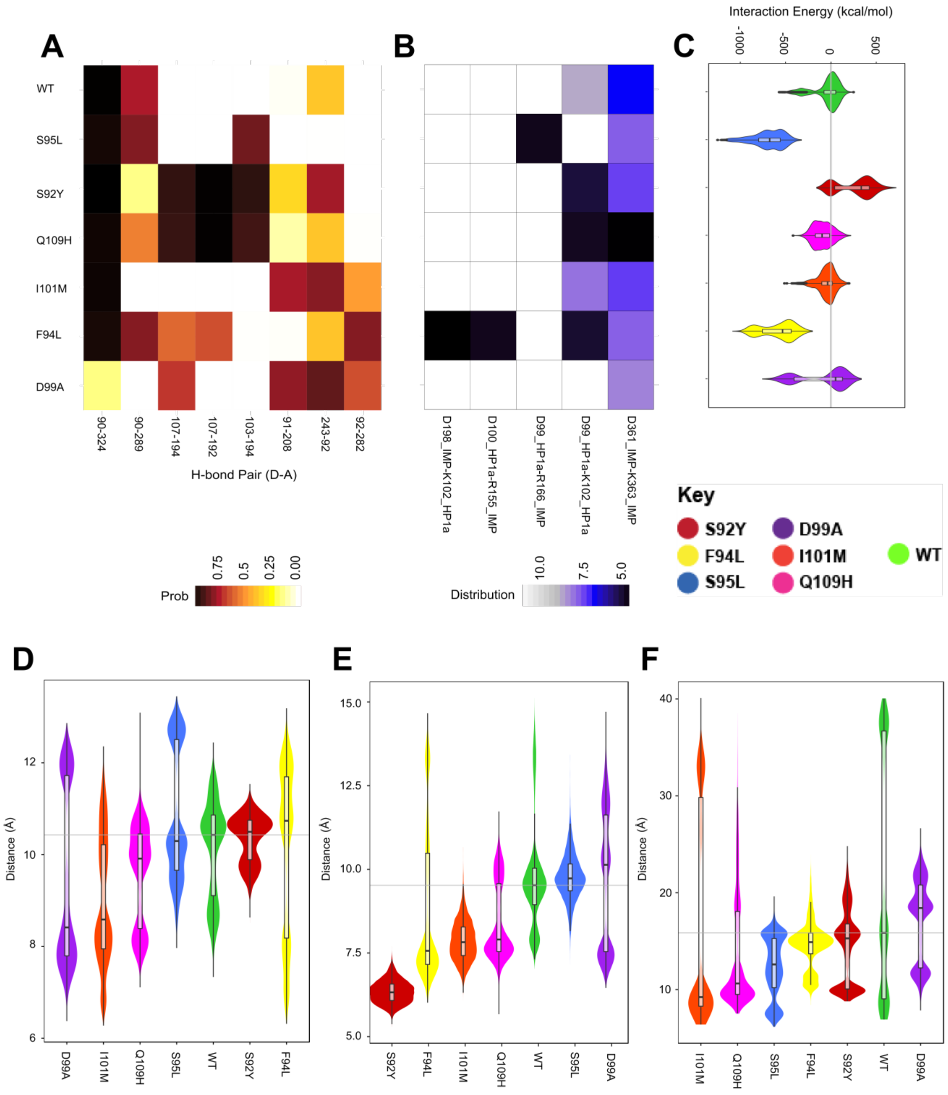

Figure S4: Genomic variants in the HP1α linker promote reorganizing of the intermolecular contacts to stabilize the complex with IMPα. (A) Hydrogen bond matrix of hydrogen bond donor and acceptor pairs in all conditions. The constant presence (probability ≥ 0.5) or absence (probability ≤ 0.5) of a hydrogen bond is defined by the black to white color scale. **(B)** The salt-bridge matrix uses the distribution value of 7.5 as a cut-off for the presence of a salt-bridge between the donor and acceptor pair. Colors in the range of light purple to black indicate the presence of a salt-bridge. Chain A refers to IMPα and chain B represents the HP1α peptide. **(C)** Total interaction energy of the HP1α-IMPα complex across all conditions. Negative kcal/mol values represent the energy of unbinding and destabilization of the complex. Values closer to or greater than zero indicate a stable interaction. **(D-F)** Distance metrics of each residue pair as outlined in **Figure 4C**.

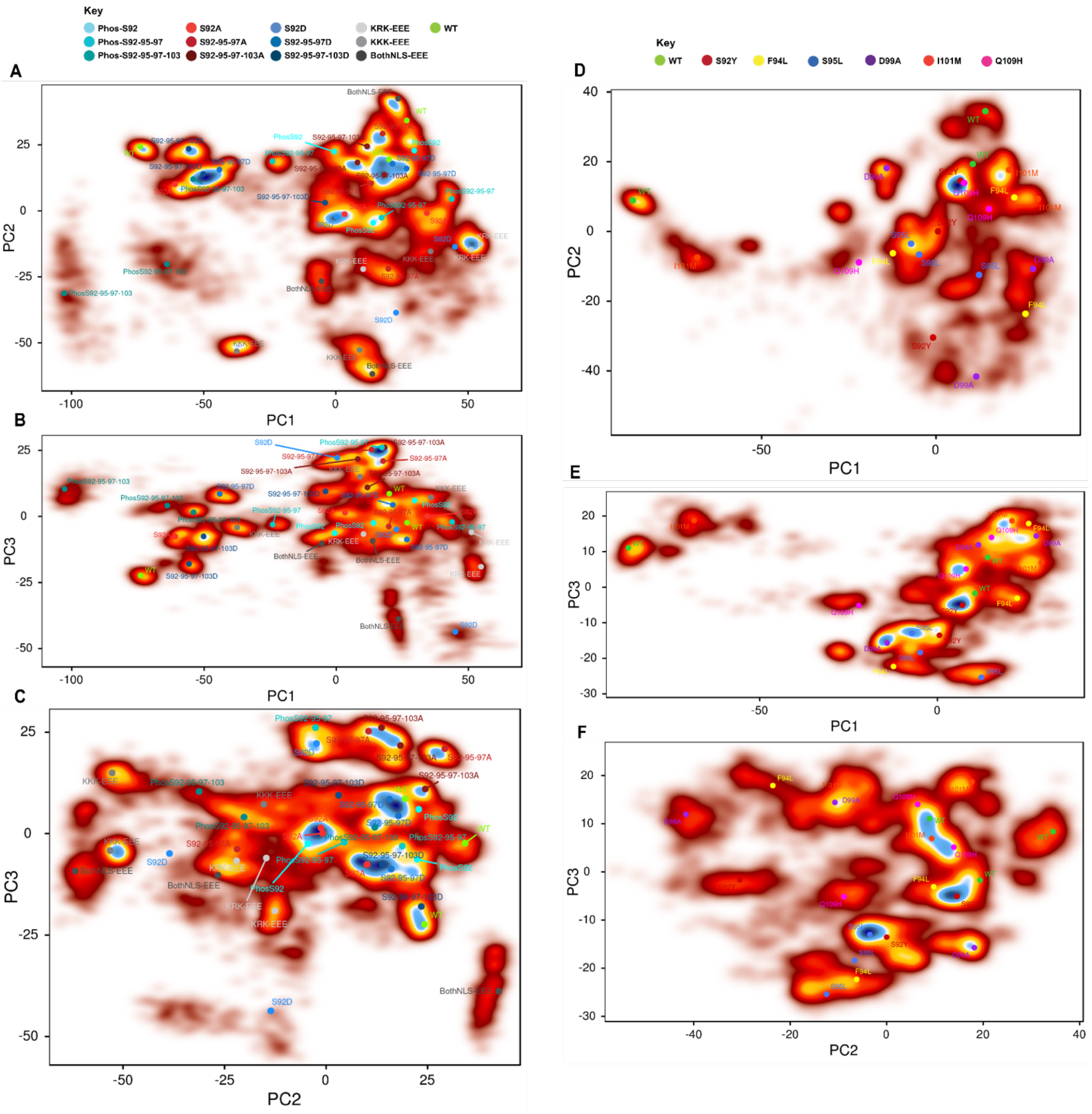

Figure S5: Phosphorylation and phosphomimetic mutation of all serine residues in the HP1α linker increases deviation from the wild-type conformation. Principle component analysis of all frames of the trajectories from each experimental condition demonstrates an overall divergence of the fully phosphorylated or phosphomimetic HP1α peptide from wild-type and all other conditions. **(A)** Comparison of PC1 and PC2 shows clustering of the wild-type, non-phosphorylatable, and partially

phosphorylated or phosphomimetic peptides. **(B)** Comparison of PC1 and PC3 illustrates the similarity via clustering together of the phosphorylation or phosphomimetic mutation of S92 through S103. **(C)** Comparison of PC2 and PC3 shows that phosphorylation of S92 through S103 diverged from all the conditions except the NLS binding mutants. **HP1α linker genomic variants are comparable to the wild-type protein.** Principle component analysis of the trajectories demonstrates clustering of the variants with wild-type with the exception of two replicate outliers from wild-type and the I101M variant. **(D)** PC1 vs PC2 **(E)** PC1 vs PC3 **(F)** PC2 vs PC3.

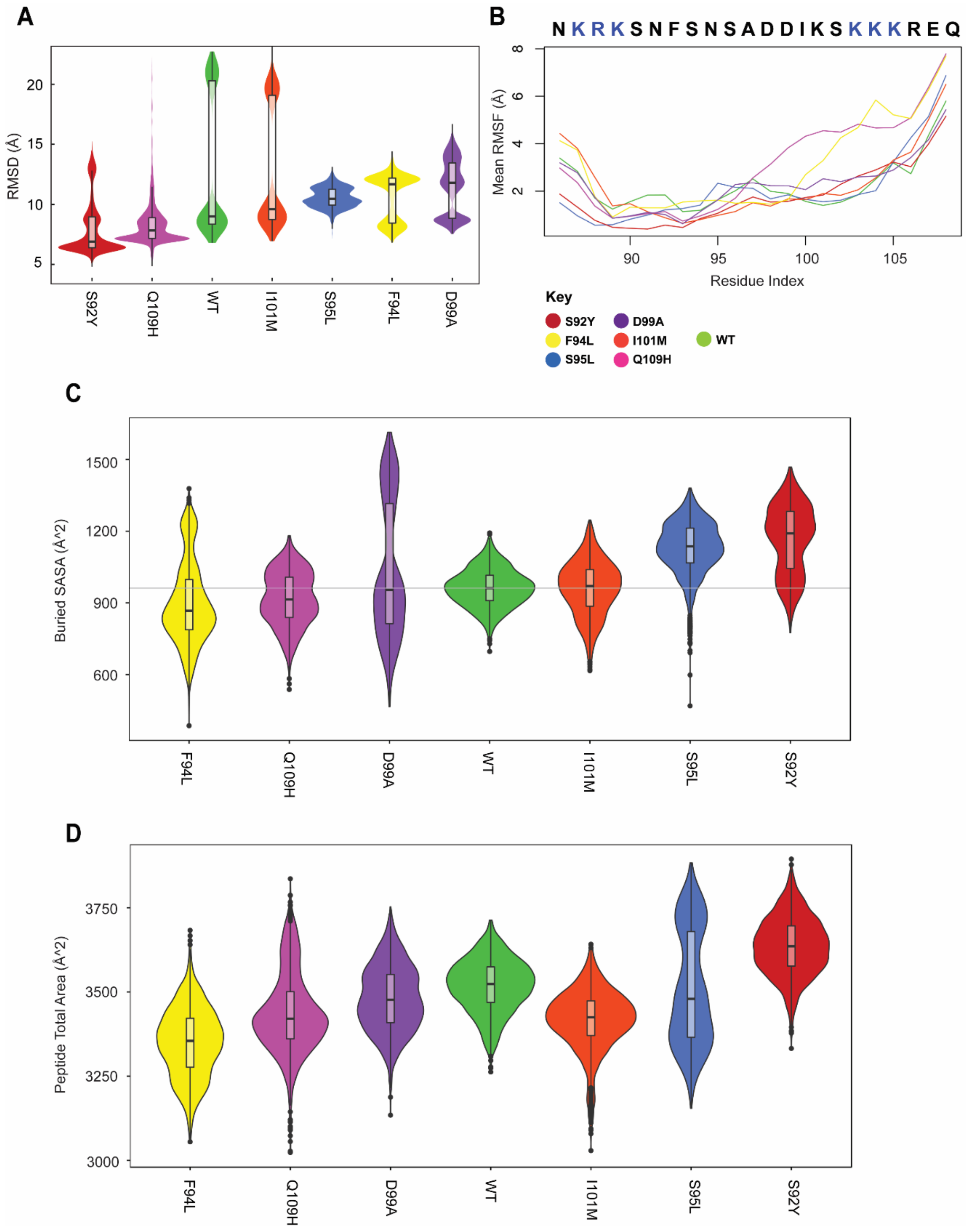

Figure S6: Genomic variants in the HP1α linker do not drastically affect the overall mobility or conformation of the peptide. (A) RMSD calculation illustrates little deviation of the variants from wild-type but increased variability similar to wild-type in the I101M and Q109H variants. **(B)** RMSF calculation shows the F94L variant diverges most from wild-type. **(C)** Buried SASA of the HP1α linker peptide in all conditions. **(D)** Total surface area of the HP1α linker peptide in all conditions.

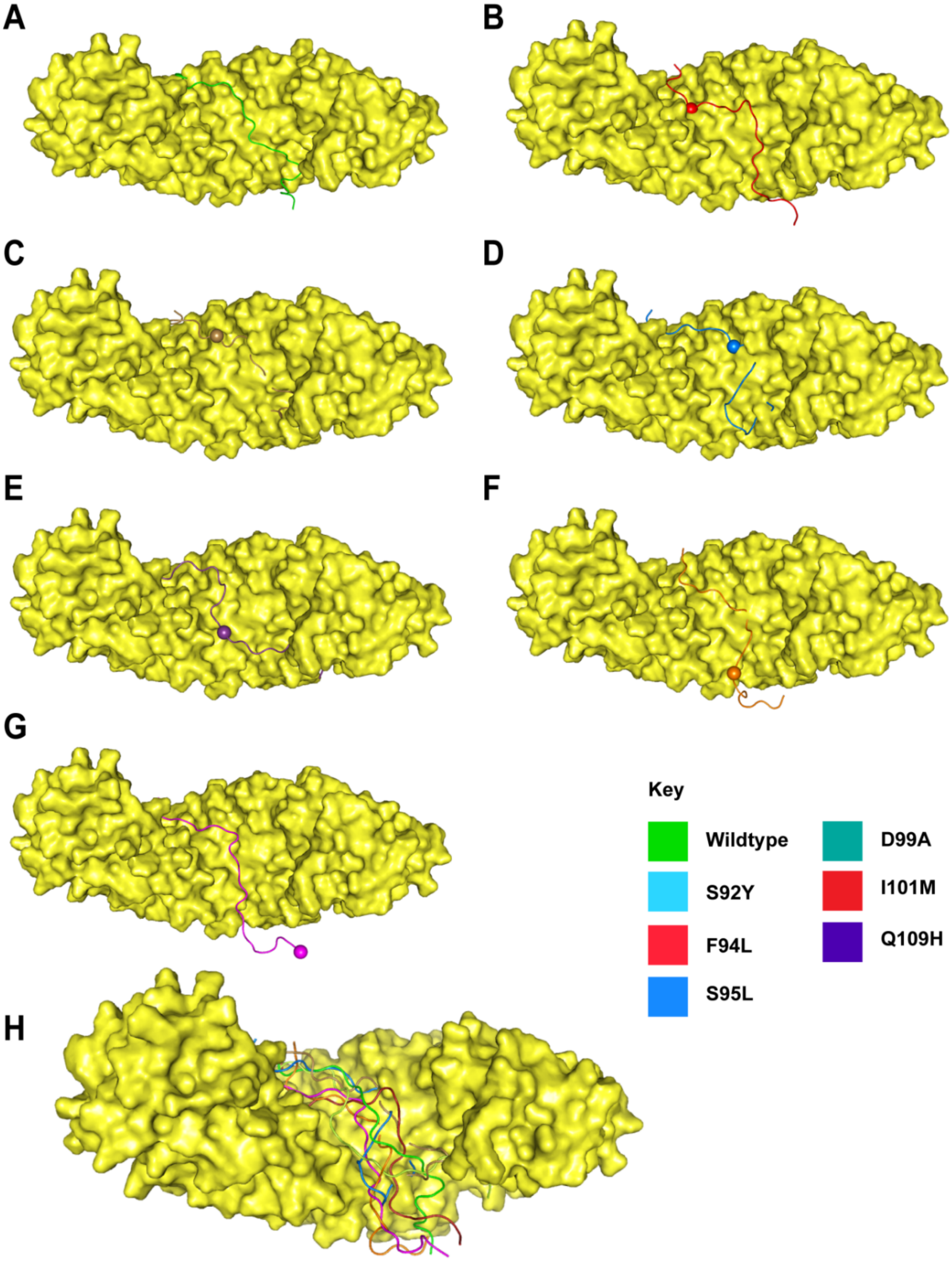

Figure S7: Visualization of the final conformation of the HP1α linker variant peptides bound to IMPα. (A) Wild-type **(B)** S92Y **(C)** F94L **(D)** S95L **(E)** D99A **(F)** I101M **(G)** Q109H **(H)** Superimposition of all peptides bound to IMPα.
